# Optimization of the GSH-Mediated Formation of Mesoporous Silica-Coated Gold Nanoclusters for NIR Light-Triggered Photothermal Applications

**DOI:** 10.3390/nano11081946

**Published:** 2021-07-28

**Authors:** Natanael Fernandes, Carolina F. Rodrigues, Duarte de Melo-Diogo, Ilídio J. Correia, André F. Moreira

**Affiliations:** 1 CICS-UBI—Health Sciences Research Centre, Universidade da Beira Interior, Av. Infante D. Henrique, 6200-506 Covilhã, Portugal; natanael.f.f.fernandes@gmail.com (N.F.); carolina.felix.rodrigues@ubi.pt (C.F.R.); demelodiogo@fcsaude.ubi.pt (D.d.M.-D.); icorreia@ubi.pt (I.J.C.); 2 CIEPQPF—Departamento de Engenharia Química, Universidade de Coimbra, Rua Sílvio Lima, 3030-790 Coimbra, Portugal

**Keywords:** cancer, gold silica shell nanoparticles, photothermal therapy, nanoclusters, glutathione

## Abstract

Cancer light-triggered hyperthermia mediated by nanomaterials aims to eliminate cancer cells by inducing localized temperature increases to values superior to 42 °C, upon irradiation with a laser. Among the different nanomaterials with photothermal capacity, the gold-based nanoparticles have been widely studied due to their structural plasticity and advantageous physicochemical properties. Herein, a novel and straightforward methodology was developed to produce gold nanoclusters coated with mesoporous silica (AuMSS), using glutathione (GSH) to mediate the formation of the gold clusters. The obtained results revealed that GSH is capable of triggering and control the aggregation of gold nanospheres, which enhanced the absorption of radiation in the NIR region of the spectra. Moreover, the produced AuMSS nanoclusters mediated a maximum temperature increase of 20 °C and were able to encapsulate a drug model (acridine orange). In addition, these AuMSS nanoclusters were also biocompatible with both healthy (fibroblasts) and carcinogenic (cervical cancer) cells, at a maximum tested concentration of 200 μg/mL. Nevertheless, the AuMSS nanoclusters’ NIR light-triggered heat generation successfully reduced the viability of cervical cancer cells by about 80%. This confirms the potential of the AuMSS nanoclusters to be applied in cancer therapy, namely as theragnostic agents.

## 1. Introduction

The development of nanomaterials for mediating the heat generation in response to laser irradiation has been widely explored in the literature as a stand-alone anticancer therapeutic approach [1,2,3]. Cancer photothermal therapy (PTT) aims to eliminate cancer cells by increasing the temperature above 42 °C through nanomaterials activation with a tumor-specific laser irradiation [4,5]. This also benefits from the nanomaterials specificity towards the tumor tissue to confine the generated heat within this area [6]. Moreover, the use of near-infrared (NIR; 750–1000 nm) light as a trigger for the photothermal therapy avoids the radiation interaction with the different biological constituents achieving a high tissue penetration [7,8]. In this way, several types of nanomaterials, such as those based on gold, carbon (e.g., carbon nanotubes, nanographene oxide), copper, tungsten, iron and molybdenum, have been under extensive study to mediate the cancer photothermal therapy [9,10,11,12,13,14]. Among them, the unique physicochemical properties of gold-based nanomaterials highly support their application as promising photothermal therapeutic agents [7,15]. These nanomaterials can present different structural organizations (e.g., spheres, rods, cages, shells and clusters) and sizes, which can be optimized to present a strong NIR absorption and high photothermal capacity by fine-tuning the localized surface plasmon resonance (LSPR) phenomenon [7,12,16,17]. The gold nanoclusters result from the gold nanospheres’ aggregation, linkage or formation in close proximity and have been explored to increase the applicability of gold nanospheres in cancer therapy [7,18,19]. Some of the most common strategies are based on the entrapment of gold nanospheres in large templates such as Albumin and PLGA-based nanoparticles or even the utilization of crosslinking spacer-arms [20,21,22]. This structural arrangement promotes the coupling of the plasmon oscillations between the adjacent gold nanospheres, which can lead to an enhanced absorption in the NIR region [23]. Additionally, recent data also demonstrated that a mesoporous silica shell can increase the photostability (i.e., prevents the degradation or reshaping) of gold nanoparticles, without impacting their photothermal potential due to the NIR light transparency [24,25,26,27]. Moreover, the silica shell mesopores also provide a reservoir for loading therapeutic agents, enabling the development of combinatorial chemo-PTT anticancer approaches [28,29,30]. Nevertheless, most of the protocols used for the production of gold nanocluster are laborious and complex involving the use of spacer-arms to attach various gold spheres, large templates to entrap the gold nanospheres, or its encapsulation in polymeric or liposomal nanoparticles [20,21,31].

Herein, a novel and straightforward methodology was developed to produce gold nanoclusters coated with a mesoporous silica shell (AuMSS) using glutathione (GSH) to mediate the aggregation of gold nanospheres. In this process, both the concentration of GSH and the source of silica (tetraethyl orthosilicate (TEOS)) were optimized, leading to the formation of several nanoaggregate formulations. The GSH is a tripeptide (cysteine, glycine and glutamic acid) with antioxidant properties and is essential for the maintenance of cellular redox homeostasis [32]. Moreover, it is well documented the gold affinity to molecules composed of thiol functional groups [33]. Therefore, the establishment of gold-thiol interactions can allow the GSH to act as a molecular bridge triggering the gold nanospheres aggregation. In fact, Chegel and coworkers reported that compounds containing amine and thiol functional groups (e.g., cysteine, urea, tris(hydroxymethyl)aminomethane and ethanolamine) can trigger the aggregation of gold nanoparticles [34]. Similarly, Stobiecka and colleagues also demonstrated that the interaction of citrate-capped gold nanoparticles with homocysteine, cysteine and GSH can lead to the formation of particles’ agglomerates [35,36,37]. Otherwise, reports available in the literature also indicate that the silica shell can also impact the physicochemical properties of gold nanoparticles [38,39]. For that purpose, the AuMSS nanoclusters’ potential for mediating the cancer PTT was assessed by determining the physicochemical properties, cytocompatibility and photothermal capacity. Moreover, the AuMSS nanoclusters drug loading capacity and release profile at pH 7.4 and 5.6 were also characterized.

## 2. Materials and Methods

### 2.1. Materials

Hydrogen tetrachloroaurate (III) trihydrate (HAuCl_4_∙3H_2_O–99.9% (metals basis), Au 49% min) was obtained from Alfa Aesar (Germany). Acridine orange (AO; >98%) was obtained from Carbosynth (Berkshire, UK). Tetraethyl orthosilicate (TEOS; >97%), tetrahydrofuran anhydrous (THF) stabilized with BHT and hexadecyltrimethylammonium bromide (CTAB; 98%) were acquired from Tokyo Chemical Industry (Tokyo, Japan). Dulbecco’s Modified Eagle medium-high glucose (DMEM-HG), Dulbecco’s Modified Eagle Medium/Nutrient Mixture F-12 (DMEM-F12), phosphate-buffered saline solution (PBS), ethanol (EtOH), Hydrochloric acid, formaldehyde, trypsin, resazurin and glutathione (GSH) reduced were purchased from Sigma-Aldrich (Sintra, Portugal). Fetal bovine serum (FBS) was obtained from Biochrom AG (Berlin, Germany). Calcein acetozxmethyl (calcein-AM) and propidium iodide (PI) were obtained from Invitrogen (Carlsbad, CA, USA). Human negroid cervix epithelioid carcinoma (HeLa cells) (ATCCs CCL-2TM) were acquired from ATCC (Middlesex, UK). Primary normal human dermal fibroblast (FibH) cells were bought from Promocell (Heidelberg, Germany). Cell culture t-flasks were acquired from Orange Scientific (Braine-l’Alleud, Belgium). Double deionized and filtered water (ultrapure water) was obtained by using a Milli-Q Advantage A10 Ultrapure Water Purification System (0.22 μm filtered; 18.2 MΩ cm at 25 °C). Cell imaging plates were acquired from Ibidi GmbH (Munich, Germany).

### 2.2. Synthesis of AuMSS Nanoclusters

The synthesis of gold nanospheres was accomplished by adapting a method described by Dias and colleagues [40]. For that purpose, 1 mL of formaldehyde (3.7 wt%) was added to a round-bottom flask containing 24 mL of ultrapure water, 50 mg of CTAB, 0.8 mL of HAuCl_4_∙3H_2_O (0.05 M) and NaOH (0.5 M). After 15 min at 80 °C, the solution was centrifuged for 20 min at 14,000× *g* to recover the gold nanospheres and resuspended in ultrapure water. Then, the gold nanospheres aggregation was promoted by adding different volumes (3; 3.5; 4; 4.5; 5 mL) of GSH solution at 6.13 mg/mL to the gold nanospheres and stirring for 90 min. The resulting gold nanoclusters were recovered by centrifugation (11,000× *g* for 20 min), resuspended in ultrapure water and reacted for 2 h with 0.2 mL of TEOS (33% *v*/*v* in ethanol), 4.56 mg of CTAB and 17.5 μL NaOH (0.5 M). The AuMSS nanoclusters were defined as Formula A, B, C, D and E, for the nanomaterials produced with 3, 3.5, 4, 4.5 and 5 mL of GSH, respectively.

From the previous formulations, the two that presented the best results in TEM and UV-Vis-NIR characterization (3.5 mL and 5 mL of GSH formulations) were used to optimize the mesoporous silica shell. For that purpose, the previously described methodology was adapted by adjusting the amount of TEOS solution added during the synthesis 0.200, 0.100, or 0.050 mL (please see Table S1). Lastly, the AuMSS nanoclusters were resuspended in hydrochloric acid 7.5% *v*/*v* in ethanol, sonicated for 1 min and centrifuged (18,000× *g*, 20 min at 25 °C) to remove the CTAB [41]. After the final washing step, the AuMSS nanoclusters were resuspended in ultrapure water.

### 2.3. Characterization of Nanocarriers’ Physicochemical Properties

The structure and morphology of AuMSS nanoclusters were characterized by Transmission Electron Microscopy (TEM-Hitachi-HT7700, Tokyo, Japan), at an accelerating voltage of 80 kV. Moreover, the size and zeta potential of the AuMSS nanoclusters were evaluated using a Zetasizer Nano ZS (Malvern Instruments, Worcestershire, UK). The UV-Vis-NIR spectra were acquired at 300 nm/min scanning rate and a wavelength range from 300 to 1100 nm in a UV-Vis-NIR spectrophotometer (Thermo Scientific Evolution™ 201 Bio UV-Vis-NIR Spectrophotometer, Thermo Fisher Scientific Inc., Waltham, MA, USA). All the spectra were acquired using the AuMSS nanoclusters at a concentration of 100 µg/mL.

The drug loading capacity was evaluated by immersing the AuMSS nanoclusters in an acridine orange (AO) methanol solution for 48 h. The AO-loaded AuMSS nanoclusters were centrifuged and the AO concentration was determined by measuring the supernatant absorbance and using the following calibration curve (Abs = 0.1981C − 0.0033; R^2^ = 0.999). Subsequently, Equation (1) was used to calculate the AO encapsulation efficiency (E.E):

(1) $$E.E.\;\left(\%\right)\;=\;\frac{\left(\mathrm{Initial}\;\mathrm{drug}\;\mathrm{weight}\;-\;\mathrm{Drug}\;\mathrm{weight}\;\mathrm{in}\;\mathrm{the}\;\mathrm{supernatant}\right)}{\mathrm{Initial}\;\mathrm{drug}\;\mathrm{weight}}\;\times \;100,$$

The AO release profile was characterized at pH 7.4 and 5.6. For that purpose, Float-A-Lyzer dialysis bags were filled with a PBS solution containing AO-loaded AuMSS nanoclusters and dialyzed against PBS at 37 °C. At different time points, 1 mL of the dialysis media was recovered and replaced with fresh PBS, maintaining the volume constant during the 48 h of the experiment. Finally, the released AO was quantified by measuring the media absorbance as described above.

### 2.4. In Vitro Photothermal Measurements

The photothermal potential of AuMSS nanoclusters was evaluated in vitro upon irradiation with a NIR laser (808 nm, 1.7 W cm^−2^) for 10 min and monitoring the temperature variations using a thermocouple sensor (accuracy of 0.1 °C) [42].

### 2.5. Cytocompatibility Assay

The AuMSS nanoclusters’ cytocompatibility was assessed in both HeLa (cervical cancer cell model) and FibH (Human fibroblasts) cells using the resazurin-based assay [43]. For that purpose, HeLa or FibH cells were cultured at a density of 10,000 cells per well in 96-well plates for 48 h, at 37 °C. Then, the medium was removed and the cells were incubated with different concentrations of AuMSS nanoclusters (from 50 to 200 μg/mL). After 24, 48 and 72 h of incubation, the medium was replaced and the cells were incubated with 110 μL of resazurin 10% (*v*/*v*). The cell viability was determined by measuring the resorufin fluorescence at λex = 560 nm and λem = 590 nm. Negative control (K^−^)-cells incubated only with culture media; positive control (K^+^)-cells incubated with EtOH.

### 2.6. Characterization of the Nanoclusters Phototherapeutic Effect

The AuMSS nanoclusters NIR-triggered cytotoxic activity was determined via resazurin assay upon 1 or 3 irradiations cycles with NIR light. Briefly, HeLa cells were cultured in 96-well plates at a cell density of 10,000 cells per well. Then, HeLa cells were incubated with AuMSS nanoclusters (Formula F, G, H and I) for 6 h and submitted to 1 or 3 NIR laser (808 nm, 1.7 W cm^−2^ for 5 min) irradiation cycles. After 24 h of incubation with AuMSS nanoclusters, the HeLa cells were incubated with resazurin and the viability was assessed as described above. Negative control (K^−^)-cells incubated only with culture media; positive control (K^+^)-cells incubated with EtOH.

Additionally, the Live/Dead assay (Invitrogen, Life Technologies, Carlsbad, CA, USA) was also used to evaluate the cytotoxic capacity of the AuMSS nanoclusters. Briefly, HeLa cells were cultured on μ-Slide 8 well Ibidi imaging plates (Ibidi GmbH, Gräfelfing, Germany) and after 24 h incubated with the AuMSS nanoclusters (Formula F, G, H and I) at a concentration of 200 μg/mL. Then, the different groups were irradiated with the laser (808 nm, 1.7 W cm^−2^) for 5 min and stained with Calcein AM (live cells) and PI (dead cells). The live/death images were obtained by Confocal Laser Scanning Microscopy (CLSM; Zeiss LSM 710, Carl Zeiss AG, Jena, Germany).

### 2.7. Statistical Analysis

Statistical analysis of the obtained results was performed using GraphPad Prism v.9.0 (Trial version, GraphPad Software, CA, USA). Data are presented as the mean ± standard deviation (s.d.). One-way analysis of variance (ANOVA) with the Student–Newman–Keuls test was used to compare different groups. A value of *p* < 0.05 was considered statistically significant.

## 3. Results and Discussion

### 3.1. Evaluation of the GSH Effect on the Agglomeration of Gold Nanoparticles

**Figure 1:**
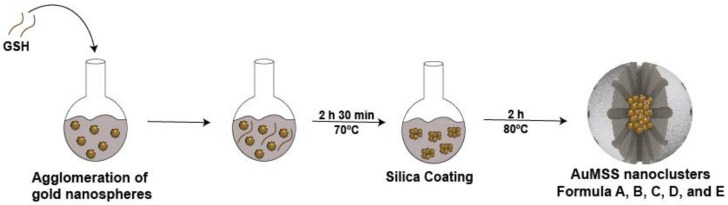
Representation of the nanoclusters’ optimization synthesis.

This work aimed to develop and optimize a new method to produce gold nanoclusters containing a mesoporous silica shell. The AuMSS nanoclusters production is divided into three main phases (as demonstrated in Figure 1 schematics): (i) synthesis of the gold nanospheres; (ii) agglomeration of gold nanospheres; and (iii) production of the mesoporous silica layer. The gold nanospheres are synthesized by promoting the reduction of gold in the presence of formaldehyde [40]. Afterward, GSH was selected to mediate the nanospheres agglomeration due to previous evidence in literature that compounds containing amine and thiol functional groups can trigger the aggregation of gold nanoparticles [34]. Finally, the mesoporous silica shell was produced via TEOS hydrolysis in the presence of CTAB.

**Figure 2:**
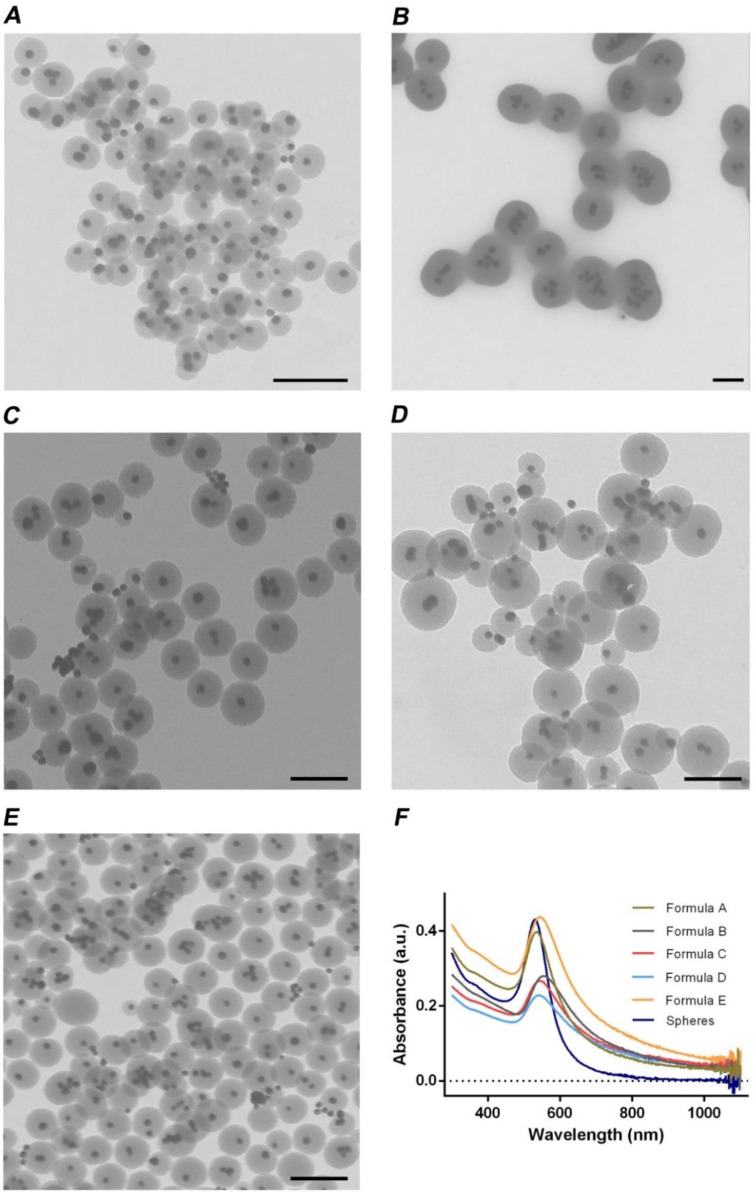
AuMSS nanoclusters morphology and UV-Vis-NIR data analysis. (**A**) TEM images of Formula A; (**B**) Formula B; (**C**) Formula C; (**D**) Formula D and (**E**) Formula E AuMSS nanoclusters. (**F**) UV-Vis-NIR spectra of different nanoformulations of AuMSS nanoclusters. Scale bar: 200 nm.

The GSH contribution to the agglomeration of gold nanospheres was studied by adding increasing amounts 3, 3.5, 4, 4.5 and 5 mL of GSH (6.13 mg/mL) to the gold nanospheres solution. Then, the newly formed gold nanoclusters were coated with mesoporous silica through the addition of TEOS (0.2 mL at 33% *v*/*v* in ethanol) resulting in the Formula A, B, C, D and E according to the volume of GSH, 3, 3.5, 4, 4.5 and 5 mL respectively. The successful agglomeration of the gold nanoparticles was preliminarily confirmed by TEM, Figure S1. The analysis of the TEM image demonstrates the successful formation of gold nanoclusters after reaction with GSH and confirmed the nanoparticles’ core-shell organization. Moreover, the TEM images also show that Formula B and E AuMSS nanoclusters present a lower quantity of non-coated gold nanoclusters (Figure 2). Apart from the morphological characterization, the UV-Vis-NIR spectra were also acquired to assess any possible alterations in the AuMSS absorption spectra. The obtained results show that the gold nanoclusters’ absorption peak suffer a slight redshift as well as an overall increase in the absorption at the 700–800 nm region when compared to gold nanospheres (Figure 2F). Furthermore, in general, an increase in the GSH amount resulted in a higher absorption in the NIR region, which is advantageous for the application of AuMSS nanocluster in PTT. Han and colleagues also reported a similar enhancement in the NIR absorption with the increase of the gold clustering on hyaluronan-based nanomaterials [44]. These changes in the gold nanoclusters absorption spectra are attributed to the coupling of the plasmon resonances due to the interaction of free electrons on the surface of particles in close proximity. Therefore, these data further confirm that the GSH can successfully mediate the aggregation of the gold nanospheres, via gold-thiol interactions and be used to promote the production of nanoclusters. Considering both TEM and UV-Vis-NIR data, Formula B and E AuMSS nanoclusters were selected as the most promising for further testings, silica shell optimization, due to the increased particle uniformity (i.e., lower number of non-coated gold clusters) and enhanced NIR absorption.

### 3.2. Optimization of the Mesoporous Silica Shell

**Figure 3:**
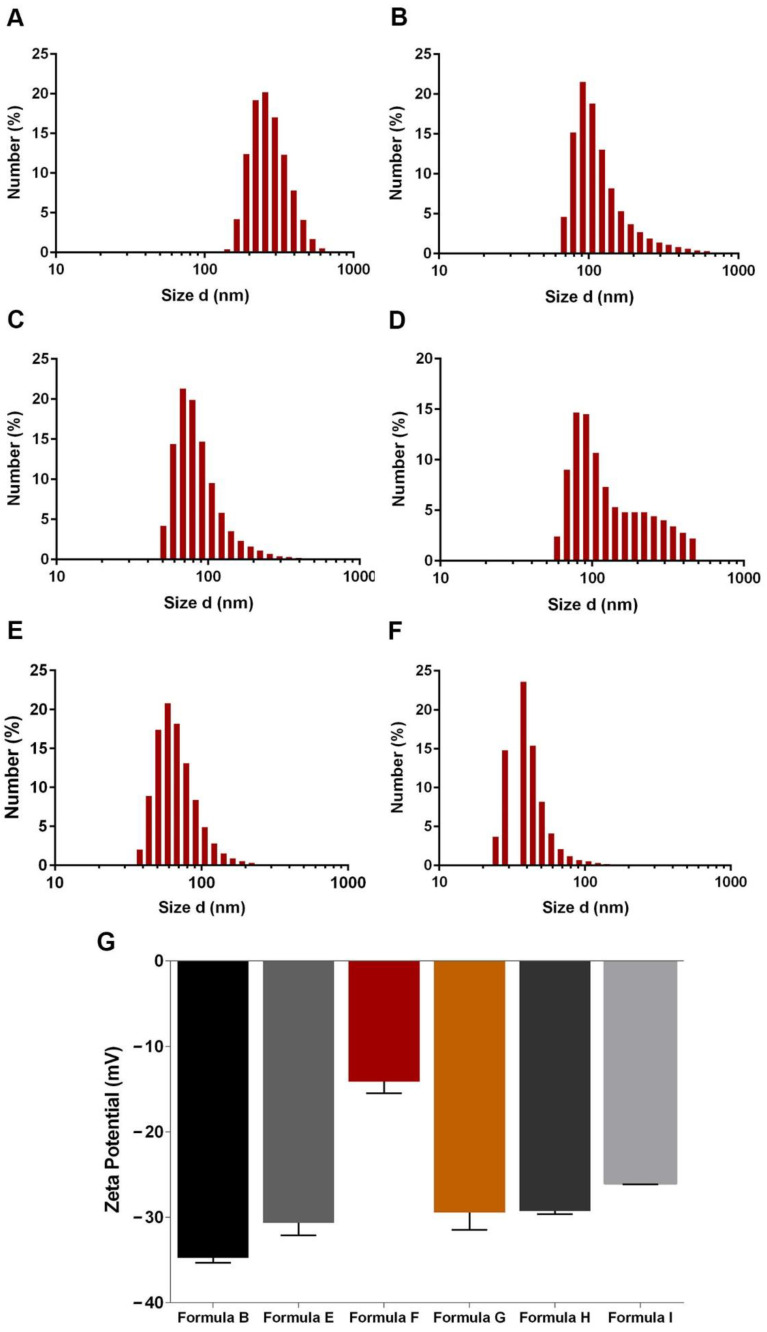
Physicochemical characterization of AuMSS nanoclusters formulations. (**A**) The size distribution of Formula B; (**B**) Formula E; (**C**) Formula F; (**D**) Formula G; (**E**) Formula H and (**F**) Formula I, *n* = 300. (**G**) Analysis of the AuMSS nanoclusters’ surface charge, *n* = 3.

The introduction of a mesoporous silica shell can also impact the physicochemical properties of the gold nanoparticles [45]. With that in mind, the mesoporous silica coating was optimized by fine-tuning the TEOS concentration. For that purpose, gold nanoclusters produced using 3.5 or 5 mL of GSH (6.13 mg/mL) were reacted with 0.200, 0.100, or 0.50 mL of TEOS (33% *v*/*v* in ethanol). The Formula B, G and F refer to the AuMSS nanoclusters produced using 3.5 mL of GSH (6.13 mg/mL) and 0.200, 0.100, or 0.50 mL of TEOS, respectively, whereas the Formula E, I and H represent the ones resulting from 5 mL of GSH (6.13 mg/mL) and 0.200, 0.100, or 0.50 mL of TEOS, respectively. The produced Formula B, E, F, G, H and I AuMSS nanoclusters were characterized by DLS to assess their size and particle distribution, Figure 3. The AuMSS nanoclusters have a homogeneous population and the reduction in the TEOS amount lead to a decrease in the nanoparticles’ mean size. The AuMSS nanoclusters produced with 3.5 mL of GSH presented a mean diameter of 267.74, 202.86 and 136 nm for Formula B, F and G, respectively. Otherwise, Formula E, H and I nanoclusters presented a mean diameter of 109.20, 91.58 and 89.67 nm, respectively. This size reduction within each nanocluster group might be justified by the reduction of the mesoporous silica shell thickness in response to the decrease in the silica source, i.e., TEOS, during the synthesis process. Additionally, this TEOS optimization also originates nanomaterials with size within the range considered ideal for intravenous administration (i.e., 100 to 200 nm), which is crucial for exploiting the leaky structure of the tumoral vasculature, i.e., the enhanced permeability and retention (EPR) effect [45,46]. Otherwise, surface charge measurements showed that all the different formulations present a negative surface charge (Figure 3G). This negative surface charge is characteristic of silica-based materials due to the silanol groups available at the surface of the AuMSS nanoclusters [47,48]. Additionally, the results are also indicative of the successful removal of the cationic CTAB molecules used both as structure-directing agents for the pores formation and surfactant for maintaining the stability of the particles [49,50]. Furthermore, nanomaterials with slightly negative surface charges often exhibit increased blood circulation times [51].

### 3.3. In Vitro Evaluation of the AuMSS Nanoclusters Photothermal Capacity

**Figure 4:**
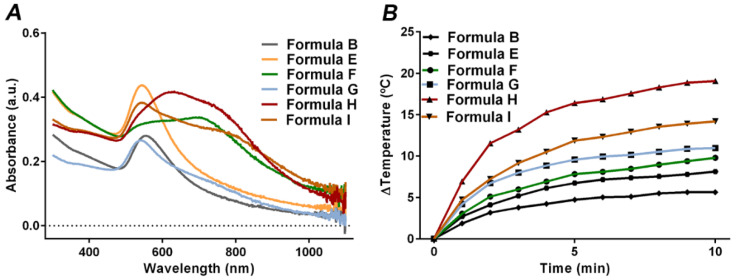
Characterization of the PTT capacity of AuMSS nanoclusters. (**A**) UV-Vis-NIR spectra of different nanoformulations of AuMSS nanoclusters; (**B**) Temperature variation curves of AuMSS nanoclusters in ultrapure water, NIR laser (808 nm, 1.7 W cm^−2^) irradiation for 10 min. Data are presented as mean ± s.d., *n* = 3.

The AuMSS nanoclusters’ ability to act as a photothermal agent was initially evaluated by measuring the UV-Vis-NIR absorption of Formula B, E, F, G, H and I (Figure 4A). As previously observed, the organization of gold nanospheres into nanoclusters induce a redshift in the absorption peak and enhance the overall absorption at the 700–800 nm region. Particularly, Formula G, H and I nanoclusters have a higher absorption in the 500 to 800 nm region with a broad absorption peak. Moreover, the obtained data also show that decreasing the TEOS amount during the AuMSS nanocluster synthesis further increases the NIR absorption. The analysis of the absorption spectra revealed that in the AuMSS nanoclusters produced with 3.5 mL of GSH, the TEOS reduction from 0.200 to 0.100 and 0.050 mL (Formula B, G and F) enhanced the absorption at 808 nm in 403 and 154%, respectively. Similar results were observed in the AuMSS nanoclusters produced with 5 mL of GSH with a 363 and 311% increase in the absorption at 808 nm for the Formula H and I, respectively. Considering the different formulations of AuMSS nanoclusters, it is possible to conclude that Formula H AuMSS nanoclusters (i.e., 5 mL of GSH and 0.050 mL of TEOS) are the ones presenting the highest NIR absorption. Nevertheless, these data support the applicability of the AuMSS nanoclusters in PTT applications.

The AuMSS nanoclusters’ photothermal capacity was investigated by recording the temperature changes in response to the nanomaterials’ irradiation with NIR light (808 nm, 1.7 W cm^−2^) for 10 min (Figure 4B). The obtained results demonstrated that all the AuMSS nanoclusters can generate heat and increase the temperature of the surrounding media in response to the NIR laser. In this regard, Formula B and E (0.200 mL of TEOS) induced the smallest increase in temperature, i.e., ΔT ≈ 8 °C. Otherwise, the NIR laser irradiation of the Formula H and I AuMSS nanoclusters resulted in the highest temperature increase, ΔT ≈ 20 and 13 °C, respectively. In turn, the Formula F and G AuMSS nanoclusters promoted an increase in the temperature of ≈11 °C. Despite these differences, all AuMSS nanoclusters presented similar heating profiles, with a pronounced rise in the temperature in the first 5 min of irradiation followed by a temperature stabilization until the end of the experiment. It is worth to note that the differences observed in the heating capacity of the different AuMSS nanoclusters are in accordance with the results previously obtained in the absorption spectra. More importantly, the heat generated in response to the NIR laser indicates that these AuMSS nanoclusters may induce the death of cancer cells through the cell membrane’s disruption, DNA damages and alterations on the metabolic pathways [6].

### 3.4. AuMSS Nanoclusters Cytocompatibility

The cytocompatibility of AuMSS nanoclusters was evaluated both on FibH and HeLa cells (Figure S2). For that purpose, the resazurin assay was used to evaluate the impact of the AuMSS nanoclusters (concentrations ranging from 50 up to 200 μg/mL) on the cellular viability after 24, 48 and 72 h. Figure S2 shows that the AuMSS nanoclusters formulations are biocompatible with both cell lines, cell viability superior to 70%, even at the highest tested concentration of 200 μg/mL. Therefore, these data further support the effectiveness of the purification step and consequent removal of CTAB from the AuMSS nanoclusters. It is worth noticing that the CTAB is highly cytotoxic, even more than doxorubicin (an anticancer drug with a broad spectrum of activity) [52,53]. Moreover, this cytocompatibility profile is in concordance with other reports for nanomedicines based on gold core-silica shell-nanoparticles [54,55].

### 3.5. Photothermal Effect Mediated by AuMSS Nanoclusters

**Figure 5:**
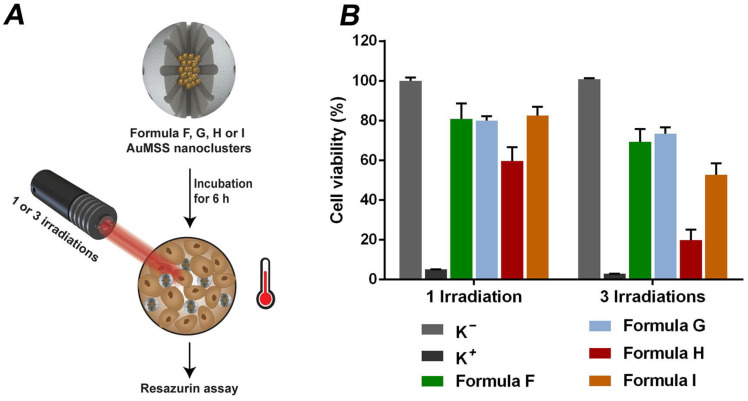
AuMSS nanoclusters cytotoxic profile towards HeLa cells. (**A**) Schematic representation of AuMSS nanoclusters cytotoxic activity upon NIR irradiation (808 nm, 1.7 W cm^−2^, 5 min); (**B**) Cytotoxic activity of different nanoclusters at 200 µg/mL with 1 and 3 laser irradiations.

The Formula F, G, H and I were selected to evaluate the PTT potential of AuMSS nanoclusters since these formulations presented the best results in the in vitro photothermal measurements. The AuMSS nanoclusters cytotoxic profile towards HeLa cells was assessed upon 1 or 3 cycles of irradiation with NIR light (Figure 5). The obtained results show that the heat generated by the AuMSS nanoclusters in response to the NIR laser irradiation leads to the reduction of the HeLa cells’ viability. Moreover, the increase in the number of NIR laser irradiation cycles further enhanced the cytotoxic effect of AuMSS nanoclusters. It is worth noticing that the highest reduction in the HeLa cells viability was achieved with Formula H and I AuMSS nanoclusters, reaching viabilities inferior to 25% and 60% after 3 cycles of irradiation, respectively. These data are in agreement with the in vitro photothermal measurements, where these two formulations mediated the highest increase in the media temperature. Hyperthermia treatments that reach temperatures above 45 °C can induce the death of cancer cells by causing irreversible damages to DNA, protein denaturation and the rupture of cell membranes [56].

**Figure 6:**
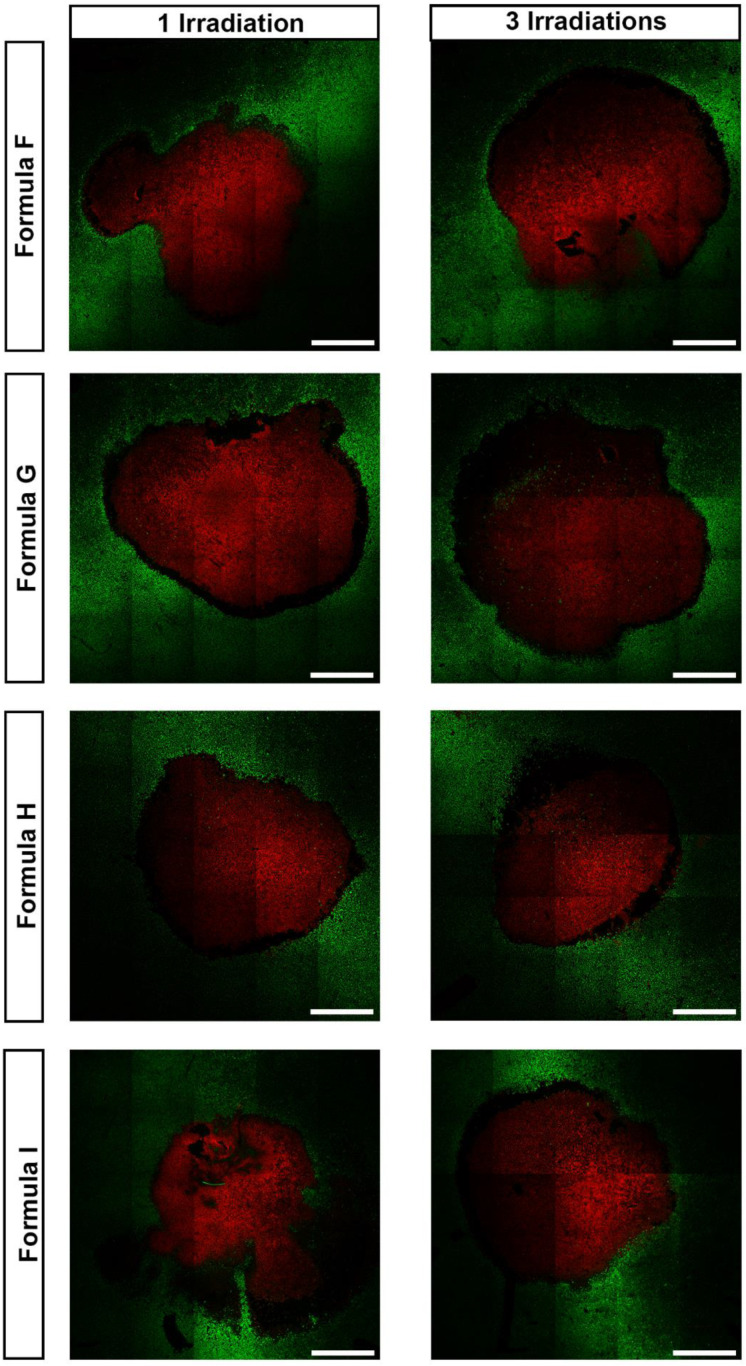
Cytotoxic effect of AuMSS nanoformulations in HeLa cells. Live/Dead CLSM images of the cytotoxic activity of different nanoclusters at 200 µg/mL with 1 and 3 laser irradiations. Green Channel: Calcein stained cells; Red channel: PI-stained cells. Scale bar: 200 μm.

The Live/Dead assay further confirmed the AuMSS nanoclusters’ photothermal therapeutic effect. In this assay, after incubation with the different AuMSS nanocluster formulations and 1 or 3 cycles of irradiation with NIR light, the HeLa cells were stained with Calcein AM and PI. In Figure 6, it is possible to observe in the NIR laser-irradiated region a large area of dead cells (i.e., red fluorescence). Furthermore, it is possible to observe that the area of dead cells increases with the number of irradiation cycles, supporting the data obtained in the resazurin assays. Therefore, the reduction of the HeLa cells viability and the Live/Dead images confirm that the AuMSS nanoclusters can mediate an on-demand NIR light-triggered photothermal therapeutic effect.

Moreover, as demonstrated in Figure S3, the AuMSS nanoclusters can also act as drug delivery systems. Considering the mesoporous structure of AuMSS nanoclusters, Formula H and I (formulations with the best photothermal effect) were selected to evaluate the drug loading capacity using AO as a drug model. Formula H and I AuMSS nanoclusters achieved AO encapsulation efficiencies of 72 and 75%, which correspond to the encapsulation of 26 and 30 µg of AO per mg of nanoparticles, respectively. Additionally, the AuMSS nanoclusters showed a similar release profile both at pH 7.4 and 5.6, with a burst release in the initial 8 h followed by a sustained and controlled release, reaching the maximum after 48 h. These findings support the future application of these AuMSS nanoclusters in combinatorial therapy (PTT and chemotherapy), allowing the development of more effective anticancer nanomedicines.

## 4. Conclusions

The unique physicochemical and biological properties of AuMSS nanoparticles prompted the application and development of new nanoplatforms for cancer therapy. The core-shell organization of AuMSS allows the combination of the imaging and photothermal capacity of gold nanomaterials with the superior stability and drug loading ability of mesoporous silica.

With that in mind, a new methodology was herein optimized to produce AuMSSs composed of a core of gold nanoclusters, for being applied as photothermal agents in cancer therapy. The development of the AuMSS nanoclusters was based on the optimization of two main parameters, the amount of GSH (i.e., the agent responsible for mediating the gold nanospheres aggregation) and silica source (i.e., TEOS). The obtained results revealed that the increase in the nanoclusters formation and the absorption capacity in the 700–800 nm with crescent amounts of GSH. Then, the production of mesoporous silica was optimized for the two selected formulations of AuMSS nanoclusters (Formula B and E). The reduction in the silica source (from 0.200 to 0.100 and 0.050 mL) during the AuMSS nanoclusters synthesis resulted in the fine-tuning of the nanomaterials’ size to values within the range considered ideal for intravenous administration (i.e., 100 to 200 nm). Furthermore, it was also observed a significant enhancement in the absorption capacity at 808 nm, 1.5–4 times higher, in the Formula G, F, H and I AuMSS nanoclusters. These improvements resulted in higher photothermal capacities, reaching the maximum ΔT of ≈20 and 13 °C with the Formula H and I, respectively. These translated to an on-demand NIR laser-triggered photothermal effect that induced the death of HeLa cancer cells, which was further enhanced when multiple irradiations were used. Overall, these data support the development of AuMSS nanoclusters as PTT agents, particularly Formula H, which combined with the gold bioimaging and mesoporous silica drug-loading capacity can be further explored to develop a multifunctional nanomedicine with an enhanced anticancer capacity.

## Data Availability

The data presented in this article are available at request from the corresponding author.

## Supplementary Materials

The following are available online at https://www.mdpi.com/article/10.3390/nano11081946/s1, Table S1: Summary of GSH and TEOS optimization during the production of different formulations of AuMSS nanoclusters, Figure S1: TEM images of gold nanospheres aggregation, Figure S2: Evaluation of AuMSS nanoformulations’ cytocompatibility, Figure S3: Characterization of AuMSS nanoformulations’ encapsulation efficiency.

## References

[1] Cheng X., Sun R., Yin L., Chai Z., Shi H., Gao M. (2017). Light-triggered assembly of gold nanoparticles for photothermal therapy and photoacoustic imaging of tumors in vivo. Adv. Mater..

[2] Rodrigues C.F., Jacinto T.A., Moreira A.F., Costa E.C., Miguel S.P., Correia I.J. (2019). Functionalization of AuMSS nanorods towards more effective cancer therapies. Nano Res..

[3] Fernandes N., Rodrigues C.F., Moreira A.F., Correia I.J. (2020). Overview of the application of inorganic nanomaterials in cancer photothermal therapy. Biomater. Sci..

[4] Saiyed Z., Telang S., Ramchand C. (2003). Application of magnetic techniques in the field of drug discovery and biomedicine. BioMagnetic Res. Technol..

[5] Melancon M.P., Elliott A., Ji X., Shetty A., Yang Z., Tian M., Taylor B., Stafford R.J., Li C. (2011). Theranostics with multifunctional magnetic gold nanoshells: Photothermal therapy and t2* magnetic resonance imaging. Investig. Radiol..

[6] Jaque D., Maestro L.M., del Rosal B., Haro-Gonzalez P., Benayas A., Plaza J.L., Martin Rodriguez E., Garcia Sole J. (2014). Nanoparticles for photothermal therapies. Nanoscale.

[7] Gonçalves A.S., Rodrigues C.F., Moreira A.F., Correia I.J. (2020). Strategies to improve the photothermal capacity of gold-based nanomedicines. Acta Biomater..

[8] Leitão M.M., de Melo-Diogo D., Alves C.G., Lima-Sousa R., Correia I.J. (2020). Prototypic Heptamethine Cyanine Incorporating Nanomaterials for Cancer Phototheragnostic. Adv. Healthc. Mater..

[9] Chen Y., Khan A.R., Yu D., Zhai Y., Ji J., Shi Y., Zhai G. (2019). Pluronic F127-functionalized molybdenum oxide nanosheets with pH-dependent degradability for chemo-photothermal cancer therapy. J. Colloid Interface Sci..

[10] Dong H., Cao Y. (2019). Carbon Nanomaterials for Optical Bioimaging and Phototherapy. Carbon Nanomater. Bioimaging Bioanal. Ther..

[11] Estelrich J., Busquets M.A. (2018). Iron oxide nanoparticles in photothermal therapy. Molecules.

[12] Moreira A.F., Rodrigues C.F., Reis C.A., Costa E.C., Correia I.J. (2018). Gold-core silica shell nanoparticles application in imaging and therapy: A review. Microporous Mesoporous Mater..

[13] Yin S., Asakura Y. (2019). Recent research progress on mixed valence state tungsten based materials. Tungsten.

[14] Zhou M., Tian M., Li C. (2016). Copper-based nanomaterials for cancer imaging and therapy. Bioconjug. Chem..

[15] Arvizo R.R., Bhattacharyya S., Kudgus R.A., Giri K., Bhattacharya R., Mukherjee P. (2012). Intrinsic therapeutic applications of noble metal nanoparticles: Past, present and future. Chem. Soc. Rev..

[16] Reis C.A., Rodrigues C.F., Moreira A.F., Jacinto T.A., Ferreira P., Correia I.J. (2019). Development of gold-core silica shell nanospheres coated with poly-2-ethyl-oxazoline and β-cyclodextrin aimed for cancer therapy. Mater. Sci. Eng. C.

[17] Riley R.S., Day E.S. (2017). Gold nanoparticle-mediated photothermal therapy: Applications and opportunities for multimodal cancer treatment. Wiley Interdiscip. Rev. Nanomed. Nanobiotechnol..

[18] Zhao P., Liu S., Wang L., Liu G., Cheng Y., Lin M., Sui K., Zhang H. (2020). Alginate mediated functional aggregation of gold nanoclusters for systemic photothermal therapy and efficient renal clearance. Carbohydr. Polym..

[19] Lee S., Lee C., Park S., Lim K., Kim S.S., Kim J.O., Lee E.S., Oh K.T., Choi H., Youn Y.S. (2018). Facile fabrication of highly photothermal-effective albumin-assisted gold nanoclusters for treating breast cancer. Int. J. Pharm..

[20] Park S., Kim H., Lim S.C., Lim K., Lee E.S., Oh K.T., Choi H.-G., Youn Y.S. (2019). Gold nanocluster-loaded hybrid albumin nanoparticles with fluorescence-based optical visualization and photothermal conversion for tumor detection/ablation. J. Control. Release.

[21] Iodice C., Cervadoro A., Palange A., Key J., Aryal S., Ramirez M.R., Mattu C., Ciardelli G., O’Neill B.E., Decuzzi P. (2016). Enhancing photothermal cancer therapy by clustering gold nanoparticles into spherical polymeric nanoconstructs. Opt. Lasers Eng..

[22] Li H., Wang P., Deng Y., Zeng M., Tang Y., Zhu W.-H., Cheng Y. (2017). Combination of active targeting, enzyme-triggered release and fluorescent dye into gold nanoclusters for endomicroscopy-guided photothermal/photodynamic therapy to pancreatic ductal adenocarcinoma. Biomaterials.

[23] Jain P.K., El-Sayed M.A. (2007). Universal scaling of plasmon coupling in metal nanostructures: Extension from particle pairs to nanoshells. Nano Lett..

[24] Kesse S., Boakye-Yiadom K.O., Ochete B.O., Opoku-Damoah Y., Akhtar F., Filli M.S., Asim Farooq M., Aquib M., Maviah Mily B.J., Murtaza G. (2019). Mesoporous silica nanomaterials: Versatile nanocarriers for cancer theranostics and drug and gene delivery. Pharmaceutics.

[25] Hanafi-Bojd M.Y., Jaafari M.R., Ramezanian N., Xue M., Amin M., Shahtahmassebi N., Malaekeh-Nikouei B. (2015). Surface functionalized mesoporous silica nanoparticles as an effective carrier for epirubicin delivery to cancer cells. Eur. J. Pharm. Biopharm..

[26] Montoto A.H., Montes R., Samadi A., Gorbe M., Terrés J.M., Cao-Milan R., Aznar E., Ibanez J., Masot R., Marcos M.D. (2018). Gold nanostars coated with mesoporous silica are effective and nontoxic photothermal agents capable of gate keeping and laser-induced drug release. ACS Appl. Mater. Interfaces.

[27] Guimarães R.S., Rodrigues C.F., Moreira A.F., Correia I.J. (2020). Overview of stimuli-responsive mesoporous organosilica nanocarriers for drug delivery. Pharmacol. Res..

[28] Moreira A.F., Rodrigues C.F., Reis C.A., Costa E.C., Ferreira P., Correia I.J. (2018). Development of poly-2-ethyl-2-oxazoline coated gold-core silica shell nanorods for cancer chemo-photothermal therapy. Nanomedicine.

[29] Hernández-Montoto A., Gorbe M., Llopis-Lorente A., Terrés J.M., Montes R., Cao-Milán R., De Greñu B.D., Alfonso M., Orzaez M., Marcos M.D. (2019). A NIR light-triggered drug delivery system using core–shell gold nanostars–mesoporous silica nanoparticles based on multiphoton absorption photo-dissociation of 2-nitrobenzyl PEG. Chem. Commun..

[30] Song Z., Liu Y., Shi J., Ma T., Zhang Z., Ma H., Cao S. (2018). Hydroxyapatite/mesoporous silica coated gold nanorods with improved degradability as a multi-responsive drug delivery platform. Mater. Sci. Eng. C.

[31] Tao Y., Li M., Kim B., Auguste D.T. (2017). Incorporating gold nanoclusters and target-directed liposomes as a synergistic amplified colorimetric sensor for HER2-positive breast cancer cell detection. Theranostics.

[32] Bansal A., Simon M.C. (2018). Glutathione metabolism in cancer progression and treatment resistance. J. Cell Biol..

[33] Xue Y., Li X., Li H., Zhang W. (2014). Quantifying thiol–gold interactions towards the efficient strength control. Nat. Commun..

[34] Chegel V., Rachkov O., Lopatynskyi A., Ishihara S., Yanchuk I., Nemoto Y., Hill J.P., Ariga K. (2012). Gold nanoparticles aggregation: Drastic effect of cooperative functionalities in a single molecular conjugate. J. Phys. Chem. C.

[35] Stobiecka M., Deeb J., Hepel M. (2010). Ligand exchange effects in gold nanoparticle assembly induced by oxidative stress biomarkers: Homocysteine and cysteine. Biophys. Chem..

[36] Stobiecka M., Coopersmith K., Hepel M. (2010). Resonance elastic light scattering (RELS) spectroscopy of fast non-Langmuirian ligand-exchange in glutathione-induced gold nanoparticle assembly. J. Colloid Interface Sci..

[37] Hepel M., Stobiecka M. (2012). Detection of oxidative stress biomarkers using functional gold nanoparticles. Fine Particles in Medicine and Pharmacy.

[38] Zhang B., Wei L., Chu Z. (2019). Development of indocyanine green loaded Au@ Silica core shell nanoparticles for plasmonic enhanced light triggered therapy. J. Photochem. Photobiol. A Chem..

[39] Fernández-López C., Mateo-Mateo C., Alvarez-Puebla R.A., Pérez-Juste J., Pastoriza-Santos I., Liz-Marzán L.M. (2009). Highly controlled silica coating of PEG-capped metal nanoparticles and preparation of SERS-encoded particles. Langmuir.

[40] Dias D.R., Moreira A.F., Correia I.J. (2016). The effect of the shape of gold core–mesoporous silica shell nanoparticles on the cellular behavior and tumor spheroid penetration. J. Mater. Chem. B.

[41] Rodrigues C.F., Reis C.A., Moreira A.F., Ferreira P., Correia I.J. (2019). Optimization of gold core-mesoporous silica shell functionalization with TPGS and PEI for cancer therapy. Microporous Mesoporous Mater..

[42] Moreira A.F., Rodrigues C.F., Jacinto T.A., Miguel S.P., Costa E.C., Correia I.J. (2020). Poly (vinyl alcohol)/chitosan layer-by-layer microneedles for cancer chemo-photothermal therapy. Int. J. Pharm..

[43] O’brien J., Wilson I., Orton T., Pognan F. (2000). Investigation of the Alamar Blue (resazurin) fluorescent dye for the assessment of mammalian cell cytotoxicity. Eur. J. Biochem..

[44] Han H.S., Choi K.Y., Lee H., Lee M., An J.Y., Shin S., Kwon S., Lee D.S., Park J.H. (2016). Gold-Nanoclustered Hyaluronan Nano-Assemblies for Photothermally Maneuvered Photodynamic Tumor Ablation. ACS Nano.

[45] Moreira A.F., Dias D.R., Correia I.J. (2016). Stimuli-responsive mesoporous silica nanoparticles for cancer therapy: A review. Microporous Mesoporous Mater..

[46] Li S.-D., Huang L. (2008). Pharmacokinetics and biodistribution of nanoparticles. Mol. Pharm..

[47] Nairi V., Medda S., Piludu M., Casula M.F., Vallet-Regi M., Monduzzi M., Salis A. (2018). Interactions between bovine serum albumin and mesoporous silica nanoparticles functionalized with biopolymers. Chem. Eng. J..

[48] Li M.Q., Lao Y.H., Mintz R.L., Chen Z.G., Shao D., Hu H.Z., Wang H.X., Tao Y., Leong K.W. (2019). A multifunctional mesoporous silica-gold nanocluster hybrid platform for selective breast cancer cell detection using a catalytic amplification-based colorimetric assay. Nanoscale.

[49] Jacinto T.A., Rodrigues C.F., Moreira A.F., Miguel S.P., Costa E.C., Ferreira P., Correia I.J. (2020). Hyaluronic acid and vitamin e polyethylene glycol succinate functionalized gold-core silica shell nanorods for cancer targeted photothermal therapy. Colloids Surf. B Biointerfaces.

[50] Zhao P., Li N., Astruc D. (2013). State of the art in gold nanoparticle synthesis. Coord. Chem. Rev..

[51] Mero A., Pasut G., Dalla Via L., Fijten M.W., Schubert U.S., Hoogenboom R., Veronese F.M. (2008). Synthesis and characterization of poly (2-ethyl 2-oxazoline)-conjugates with proteins and drugs: Suitable alternatives to PEG-conjugates?. J. Control. Release.

[52] He Q., Gao Y., Zhang L., Zhang Z., Gao F., Ji X., Li Y., Shi J. (2011). A pH-responsive mesoporous silica nanoparticles-based multi-drug delivery system for overcoming multi-drug resistance. Biomaterials.

[53] Jia Y.P., Shi K., Liao J.F., Peng J.R., Hao Y., Qu Y., Chen L.J., Liu L., Yuan X., Qian Z.Y. (2020). Effects of Cetyltrimethylammonium Bromide on the Toxicity of Gold Nanorods both In Vitro and In Vivo: Molecular Origin of Cytotoxicity and Inflammation. Small Methods.

[54] Zeng Q., Zhang Y., Ji W., Ye W., Jiang Y., Song J. (2014). Inhibitation of cellular toxicity of gold nanoparticles by surface encapsulation of silica shell for hepatocarcinoma cell application. ACS Appl. Mater. Interfaces.

[55] Chan M.-H., Lin H.-M. (2015). Preparation and identification of multifunctional mesoporous silica nanoparticles for in vitro and in vivo dual-mode imaging, theranostics, and targeted tracking. Biomaterials.

[56] De Melo-Diogo D., Pais-Silva C., Dias D.R., Moreira A.F., Correia I.J. (2017). Strategies to improve cancer photothermal therapy mediated by nanomaterials. Adv. Healthc. Mater..

