# Supplementary material for: Optimization of the GSH-Mediated Formation of Mesoporous Silica-Coated Gold Nanoclusters for NIR Light-Triggered Photothermal Applications
Source: Nanomaterials (Basel). 2021 Jul 28;11(8):1946. doi: 10.3390/nano11081946 (PMC8401642; doi:10.3390/nano11081946)
Supplement: Supplementary file 1 [file nanomaterials-11-01946-s001.zip › nanomaterials-1289614-supplementary.pdf]

Supporting Information

Optimization of the GSH-Mediated Formation of Mesoporous Silica-Coated Gold Nanoclusters for NIR Light-Triggered Photothermal Applications

Natanael Fernandes[1], Carolina F. Rodrigues[1], Duarte de Melo-Diogo[1], André F. Moreira[1*], Ilídio J. Correia[1,2*]

[1] CICS-UBI – Health Sciences Research Centre, Universidade da Beira Interior, Av. Infante D. Henrique, 6200-506 Covilhã, Portugal.

[2] CIEPQF — Departamento de Engenharia Química, Universidade de Coimbra, Rua Sílvio Lima, 3030-790 Coimbra, Portugal.

[*] Corresponding author. Tel.: +351 275 329 002; Fax: +351 275 329 099; e-mail: afmoreira@fcsaude.ubi.pt and icorreia@ubi.pt.

1. Methods

1.1 Production of gold nanoclusters

Table S1: Summary of GSH and TEOS optimization during the production of different formulations of AuMSS nanoclusters.

<table>
<tr><th>Formula</th><th>GSH (mL)</th><th>TEOS (mL)</th></tr>
<tr><td>A</td><td>3</td><td rowspan="5">0.200</td></tr>
<tr><td>B</td><td>3.5</td></tr>
<tr><td>C</td><td>4</td></tr>
<tr><td>D</td><td>4.5</td></tr>
<tr><td>E</td><td>5</td></tr>
<tr><td>F</td><td rowspan="2">3.5</td><td>0.50</td></tr>
<tr><td>G</td><td>0.100</td></tr>
<tr><td>H</td><td rowspan="2">5</td><td>0.50</td></tr>
<tr><td>I</td><td>0.100</td></tr>
</table>

2. Results

2.1. Evaluation of the GSH effect on the agglomeration of gold nanoparticles

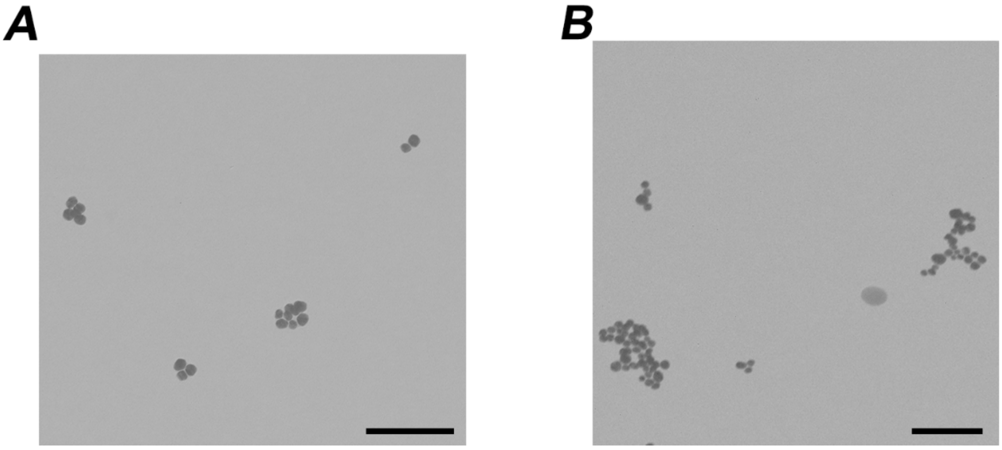

Figure S1: TEM images of gold nanospheres aggregation. 3.5 mL (A) and 5 mL (B) of GSH. Scale bar: 200 nm.

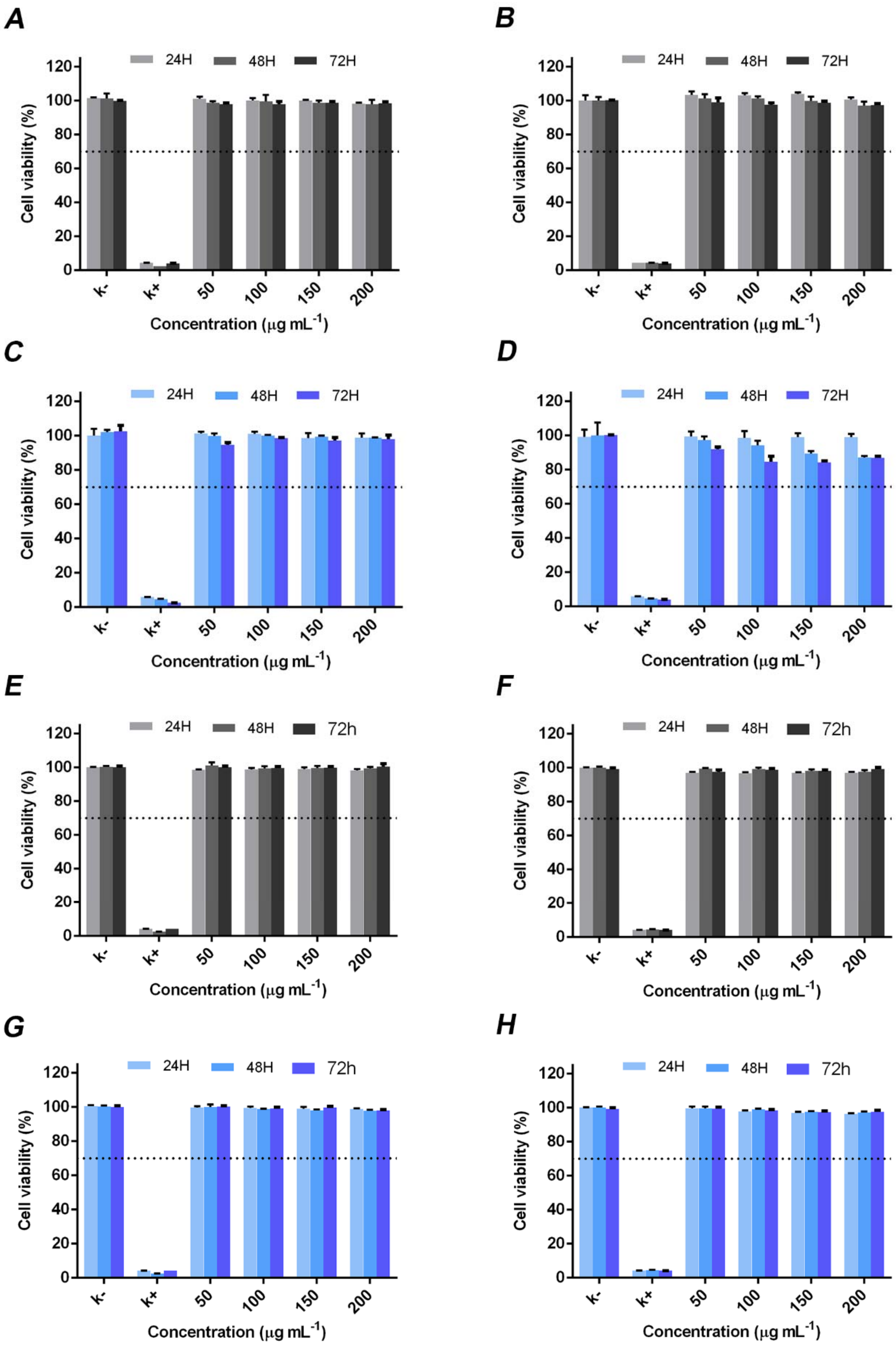

Figure S2: Evaluation of AuMSS nanoformulations' cytocompatibility at 24, 48, and 72 h on HeLa (A, C, E, and G) and FibH cells (B, D, F, and H). Cytocompatibility analysis for AuMSS nanoclusters of Formula B (A) and (B), Formula E (C) and (D), Formula H (E).

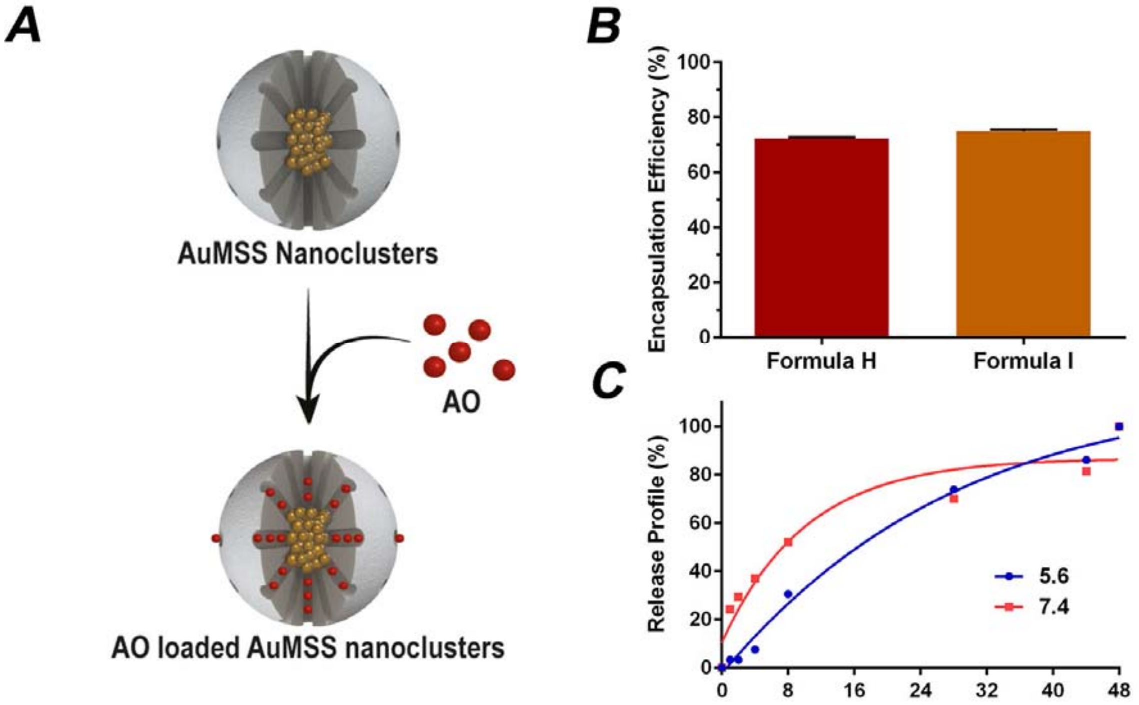

Figure S3: Characterization of AuMSS nanoformulations' encapsulation efficiency. (A) Schematics of AO loading in AuMSS nanoclusters. (B) AO encapsulation efficiency in AuMSS nanoclusters (Formula H and I).
